# A National Survey of Community Pharmacists on Smoking Cessation Services in Thailand †

**DOI:** 10.3390/pharmacy6030101

**Published:** 2018-09-17

**Authors:** Surarong Chinwong, Dujrudee Chinwong

**Affiliations:** Department of Pharmaceutical Care, Faculty of Pharmacy, Chiang Mai University, Chiang Mai 50200, Thailand; surarong@gmail.com

**Keywords:** smoking cessation, community pharmacists, tobacco control

## Abstract

Providing smoking cessation services is one role of community pharmacists in Thailand. This cross-sectional study aimed to investigate activities and barriers related to smoking cessation services provided in community pharmacies in Thailand, as well as to compare these activities and barriers between those pharmacists providing and those not providing smoking cessation services. A postal questionnaire was conducted to collect information from community pharmacists across Thailand. In all, 413 valid responses were received from 5235 questionnaires, giving a 7.9% response rate. Of the 413 respondents, 152 (37%) pharmacists provided smoking cessation services in their pharmacy. The activities of smoking cessation services varied. Time for counseling each smoker varied, a mean of 15.1 ± 10.9 min (range 1–60) per person for the first time, and 8.9 ± 6.7 min (range 1–30) for each follow-up visit. Community pharmacists, providing smoking cessation services, were more likely to have pharmacist assistants, be a member of the Thai Pharmacy Network for Tobacco Control, and have more than 1 pharmacist on duty. The most dispensed pharmaceutical product for smoking cessation was nicotine gum. Their most perceived barriers were being unable to follow-up and inadequate staff. In conclusion, only a minority of community pharmacists in Thailand are engaged in smoking cessation activities, even though some perceived barriers existed.

## 1. Introduction

Tobacco smoking is a preventable cause of death and causes more than six million deaths annually [1]. Smoking accounted for one in ten of all deaths in Thailand, and is related with increasing health expenditure [2]. Therefore, smoking cessation is one strategy to reduce the cause of preventable death from tobacco use. Offering help to quit tobacco use is one strategy for tobacco cessation, as recommended by the World Health Organization (WHO) [3]. Thailand has adopted the WHO policy for tobacco control, and has been recognized for its success in tobacco control for many years [4]. 

Community pharmacists play key roles in helping smokers to quit smoking [5,6]. Pharmacy professionals are in an optimal position to aid smoking cessation, due to their roles in providing counselling and smoking aid products to support cessation. It has been well established that interventions for smoking cessation by pharmacists are cost-effective [7]. As community pharmacists are in locations where patients could easily access, evidence in many countries has shown the success of smoking cessation services provided by community pharmacists [8,9]. Smoking cessation services were provided by health care workers, including pharmacists in many countries, such as in the UK, USA, Finland, as well as Thailand [5,6,8,9,10,11,12,13,14,15,16,17].

In Thailand, community pharmacies are in all provinces across the country and are primary places where people can go when they have a minor illness. Some community pharmacies provide smoking cessation services. Likewise, providing smoking cessation services in Thai community pharmacies is of interest to both professionals and patients. Community pharmacists can dispense nicotine replacement therapy (NRT) and non-nicotine products to strengthen their smoking cessation services, for those smokers needing pharmacotherapy to stop smoking. Research has confirmed that Thai community pharmacists engage in many smoking cessation services [8,10]. 

Smoking cessation services provided in community pharmacies in Thailand have been encouraged by the Thai Pharmacy Network for Tobacco Control (TPNTC) since 2004. Community pharmacists have been trained by the TPNTC using both lectures and practice. The first national survey in 2010 on community pharmacists’ roles in providing smoking cessation services, showed that the community pharmacist at least engaged in helping patients to quit smoking by offering brief advice [8]. Thananithisak et al. [10] conducted a study among 42 community pharmacists in Bangkok, to learn about the experiences of community pharmacists. They focused on pharmacists’ perceptions of their roles, barriers, and tobacco control activities. This study identified eight barriers in the provision of smoking cessation services, the most important three being: Lack of patient demand, lack of educational materials, and lack of smoking cessation products. This study did not cover community pharmacies across Thailand. Other studies in other countries, also identified barriers preventing community pharmacists from achieving the highest level of offering their practices of smoking cessation services. For example, these barriers included time constraints, no interest from smokers, lack of knowledge and skills needed to help smokers to quit, lack of confidence, inadequate staffing, and no reimbursement [8,10,18,19,20,21]. Since 2004, provision of smoking cessation by community pharmacists has increased encouraged by the TPNTC, and the pharmacy faculties at many universities have added smoking cessation content in their curriculum [22]. 

However, smoking cessation services are only available in some community pharmacies in Thailand. Thus, this study aimed to investigate activities and barriers related to smoking cessation services provided in community pharmacies in Thailand, as well as to compare these activities and barriers between those pharmacists providing and those not providing smoking cessation services. This will provide policy makers and organizations, such as the TPNTC and pharmacy faculties, with important information about increased roles of community pharmacists in promoting smoking cessation. 

## 2. Materials and Methods

### 2.1. Participants and Procedures

A cross-sectional survey was conducted among community pharmacies in Thailand, between 2013 and 2014. This study aimed to explore roles of community pharmacists in tobacco control, especially in providing smoking cessation services. The participants included community pharmacists across Thailand. A list of all community pharmacies and their addresses were received from the Thai Food and Drug Administration (Thai FDA), Ministry of Public Health, Faculty of Pharmacy, Chiang Mai University, and the Office of Pharmacy Accreditation (Thailand). Therefore, the questionnaires were sent to all community pharmacies, according to the list compiled from these organizations. 

### 2.2. Questionnaire Development and Data Collection 

The questionnaire used to collect information from participants was designed to be self-administered, and all responses were voluntary. The questionnaire was developed by the research team based on the objectives of the study and literature reviews. The content validity of the questionnaire was also reviewed by the research team and experts in tobacco control. The questionnaire was then tested with ten pharmacists, for the use of appropriate language, before sending the questionnaire to participants. The aim of this process was to ensure that the pharmacists properly understood each item in the questionnaire.

The questionnaire included general information regarding pharmacists, and information on activities of community pharmacists providing smoking cessation services. The demographic information included sex, age, length of being a community pharmacist, having a pharmacist assistant, location of the pharmacy, number of pharmacists on duty, being a member of the TPNTC, and products for smoking cessation available in their community pharmacies. The information on activities related to smoking cessation services in their community pharmacies, for those providing smoking cessation services, included number of people asking for help with smoking cessation, time spent with the smoking cessation services and services to aid smoking cessation. In addition, an open-ended question was asked about motivation of pharmacists to help smokers to quit, for those pharmacists offering smoking cessation services in their drugstores. The last part focused on perceived barriers by community pharmacists in providing smoking cessation services; this part was for all pharmacists to answer. Eleven Likert scale items (4-points: strongly agree, agree, disagree, and strongly disagree) inquired about perceived barriers for smoking cessation services. In all, 38 questions included a mix of multiple choice, open-ended, and Likert scale items.

Pharmacists providing smoking cessation services were identified by the question “Do you provide a smoking cessation service in your pharmacy?”, where a “Yes” response was classified as providing smoking cessation services; whilst a “No” was classified as not providing smoking cessation services. 

Questionnaires with cover letters explaining the purpose of the study and confidentiality were sent by post to 5450 community pharmacies, according to their addresses on the list. To increase the response rate, pharmacists were later contacted by telephone and given reminders. Unfortunately, many telephone numbers of pharmacists were incorrect or missing. 

### 2.3. Statistical Analysis

STATA Software, Version 12 (StataCorp LP, College Station, TX, USA) was used for statistical analyses. The significance level was set as two-tailed, and at *p*-value of <0.05. For descriptive statistics, means ± standard deviations for continuous variables, and frequencies with percentages for categorical variables were reported. The differences between the two groups (such as pharmacists providing services and those not), were compared using Fisher’s exact test for categorical variables, or independent *t*-test for continuous variables. For the 4-point Likert scale, data were grouped in two groups only (agree vs. disagree).

### 2.4. Ethics

This study protocol was approved by the Ethics Review Committee, Faculty of Pharmacy, Chiang Mai University, Thailand, before commencing the study. All participants were informed about the study protocol through the subject information sheet, and that their completing the questionnaire was voluntary. 

## 3. Results

### 3.1. Response Rate

From the initial questionnaires sent to 5450 pharmacists, failure delivery notifications were received for 215 (3.9%), giving a denominator of 5235 pharmacists. Of the 435 returned questionnaires, duplicate submission to the same community pharmacies (10) and incomplete questionnaires unsuitable for the analysis (10) were removed. Hence 413 questionnaires were included in the analysis, representing a 7.9% response rate (413/5235). Hence, percentages presented are based on numbers of respondents answering each question. 

### 3.2. Characteristics of the Respondents

Of the 413 community pharmacists, who answered the questionnaire, 152 (37%) reported having smoking cessation services provided in their community pharmacies. More pharmacists were women than men (60% and 40%), and the average age was 40.9 ± 12.7 years, ranging from 22 to 82. Most had graduated as a pharmacist before 1994, that is, graduated more than 20 years previously. Almost one half served as community pharmacists for less than 10 years. Most pharmacists (97%) agreed that providing smoking cessation services was one role of community pharmacists. Community pharmacists providing smoking cessation services, were more likely to have pharmacist assistants, be a member of the TPNTC, and have more than 1 pharmacist on duty. Details are shown in Table 1.

Smoking cessation products were available in 316 pharmacies (77%), and these products included nicotine gum, nicotine patch, bupropion, nortriptyline, varenicline, and herbal mixture for smoking cessation. Nicotine gum (73%) was the most dispensed pharmaceutical product to aid smoking cessation (Table 2). The most frequent activities pharmacists reported having performed for smoking cessation in their community pharmacies were providing materials to aid smokers to quit, e.g., brochures, posters, and video (Table 3). 

For those 152 pharmacists who provided smoking cessation services, an average of 3.4 ± 4.6 smokers monthly, for each pharmacy, received smoking cessation services; an average of 6.4 ± 6.9 smokers/month could stop smoking; whilst an average of 9.0 ± 9.0 smokers/month could reduce their smoking. Time spent in each cessation service was about 15 min per person for the first time, and 9 min for each follow-up visit (Table 4). 

The top two perceived barriers on providing smoking cessation services, were being unable to follow-up patients after providing the cessation services and inadequate staffing. These barriers did not significantly differ between those providing and those not providing cessation services. This study showed six significant different perceived barriers between the two groups, i.e., lack of time, lack of population demand, no smoking cessation products available in the drugstore, lack of knowledge and skills, lack of self-confidence, and lack of media/equipment. Interestingly, no payment for providing smoking cessation services was the lowest perceived barriers (23%), and did not significantly differ between the two groups. Details are shown in Table 5.

## 4. Discussion

### 4.1. Smoking Cessation Services Provided by the Community Pharmacists

This national survey across Thailand showed that approximately 37% of respondents provided smoking cessation services in their community pharmacies. A related study [8], also set in Thailand, showed a level of only 15%. In Thailand, community pharmacists are in an optimal position to provide smoking cessation services to patients, as pharmacists can be easily reached and asked for advice. However, not every community pharmacist can provide smoking cessation services, as smoking cessation requires not only knowledge and skills, but also time spent in assisting smokers to quit. This study showed that community pharmacists from all parts of Thailand engaged in providing smoking cessation services, mostly in Bangkok and northern Thailand. Community pharmacists at least had activities related to tobacco control or smoking cessation services provided to the public. These activities comprised (1) providing materials to aid smokers in quitting smoking, e.g., pamphlets, brochures, posters; (2) showing symbols in front of their pharmacies on helping smokers to quit smoking; (3) providing a designated area for smoking cessation services; and (4) engaging in community activities related to tobacco control, e.g., organizing a camp for students to learn the dangers of tobacco and to avoid smoking, and participating in the nonsmoking week in late May, as 31 May is World No Tobacco Day. Some community pharmacists were encouraging community members to reduce or stop smoking through radio broadcasts or journals in the community. Pharmacists who were members of the TPNTC were more likely to performing all four of these activities than nonmembers. A study conducted in the UK by Dewsbury et al., revealed that smoking cessation supported by community pharmacists had been viewed as being most valued by clients [9]. The results of this study showed that almost all pharmacists (97%) agreed that smoking cessation is an important role of community pharmacists. This finding is consistent with a related Thai study [10], suggesting that community pharmacists perceived they had important roles in helping smokers to quit smoking. 

Many smoking cessation aid products were available in community pharmacies: Nicotine gum, Nicotine patch, Bupropion, Nortriptyline, Varenicline, and Herbal formulas for smoking cessation. In Thailand, these products do not require a prescription, but need to be dispensed by a pharmacist. Nicotine gum was found to be incorrectly used by smokers as it is quite difficult to use, and a patient needs to know how to use it properly to get the most effectiveness from the product, to avoid adverse effects that may occur. This constitutes a very important role of pharmacist to help patients. A study by Kurko et al. in Finland found inappropriate use of NRT products for other purposes, and not for smoking cessation, such as products misused or used in harm reduction purposes [23]. Thus, pharmacists should communicate with clients or smokers concerning the proper use of NRT products for smoking cessation purposes. 

For those 152 community pharmacists who provided smoking cessation services daily, the number of smokers who stopped smoking after receiving smoking cessation services ranged from 0 to 40 individuals, for each pharmacy. Six smokers, monthly, on average, could quit smoking, and 9 smokers, monthly, could reduce their smoking. It seemed that the number of smokers receiving smoking cessation services was quite low, and some pharmacists did not provide smoking cessation services at the time the study was conducted. This implied that pharmacists should encourage and motivate their customers to participate in smoking cessation services. 

This study revealed that pharmacists not providing smoking cessation services were older, nonmembers of the TPNTC, did not have a pharmacist assistant, had only one pharmacist on duty, and did not stock smoking cessation products, compared to pharmacists providing smoking cessation services. In Thailand, levels of smoking cessation services provided by community pharmacists have been increasing in the past 10 to 15 years. “New generation” pharmacists have learned about smoking cessation services during their university study, and universities have only begun teaching this area recently [22]. TPNTC member pharmacists were trained by the TPNTC and were further interested in providing smoking cessation services. Therefore, the more recent pharmacy graduates or pharmacists who were TPNTC members were more likely to provide smoking cessation services, as compared with others. 

### 4.2. Perceived Barriers of Smoking Cessation Services by Community Pharmacists

Community pharmacists perceived some barriers preventing them from providing smoking cessation services. First, being unable to follow-up after providing the service was the most common issue considered to be a barrier, and did not differ between those pharmacists providing services and those not. Arranging follow-up was one part of the 5 A’s (Ask, Advise, Assess, Assist, and Arrange) that pharmacists should perform, and pharmacists may have thought that this entailed a difficult task due to reasons, such as no time or no interest from clients. The 5 A’s comprised asking all patients of their smoking status; advising smokers to stop smoking; assessing the willingness to quit; assisting smokers to quit; and arranging follow-up [24]. Therefore, having professional communication with community pharmacists that involved follow-up was essential to help motivate smokers to stop smoking. This included training them to understand the purpose of follow-up and how to conduct it. 

Second, inadequate staffing was another perceived barrier, and did not differ between the two groups. Community pharmacies providing smoking cessation services had more than one pharmacist on duty and pharmacist assistants. Smoking cessation services require time spent with smokers to counsel them, especially for the first time, about 15 min and about 9 min for each follow-up visit. Normally in Thailand, only one pharmacist is on duty; therefore, providing smoking cessation services without other personnel was quite difficult. This was similar to other studies that reported a lack of staff as a barrier [8,10,15,20,25]. 

This study identified five barriers more common among those pharmacists not providing smoking cessation services: Lack of demand, lack of knowledge and skills, lack of self-confidence, no smoking cessation products available in the drugstore, and lack of media/equipment or educational materials for providing smoking cessation services. This was consistent with findings of studies conducted in Australia, Qatar, and the USA, which also found that lack of patient demand was a barrier for providing public health services, i.e., smoking cessation [15,18,25]. As smoking is unacceptable in Thai society, especially female smoking, where the provision of smoking cessation services is a role of pharmacists, identifying smoking status should be an initial step in pharmacy practice. This could identify more smokers and those who could be assisted to quit. Communication with pharmacists involving asking clients’ smoking status is essential and could help smokers to quit smoking. This would also enhance the roles of pharmacists, regarding tobacco control. 

Two identified barriers in providing smoking cessation services, i.e., lack of knowledge/skills and lack of self-confidence, were also identified by other studies [10,26,27]. As these barriers may be overcome by training; the TPNTC and the pharmacy faculties of universities could usefully provide more relevant training to pharmacists and pharmacy students. 

Another perceived barrier to providing smoking cessation services was not having relevant medications, which was also identified in related studies [10,26]. However, this study found that Thai pharmacists perceived lack of smoking cessation medications as a weaker barrier, as per the findings of a related Thai study conducted in 2008 [10]. This was because medications for smoking cessation were only available in hospitals, at the time that the study was conducted. However, nicotine replacement products became available in community pharmacies starting July 2005.

This study also identified the lack of materials for smoking services as a barrier to service provision, a result consistent with a related study conducted in Thailand by Thananithisak et al. [10], and other studies [26,28]. This is despite that materials to aid smoking cessation could be requested without any charge from the Action on Smoking and Health Foundation Thailand (ASH Thailand). In Thailand, pharmacists and health personnel or people could receive educational materials or equipment for smoking cessation, such as posters, stickers, and videos from ASH Thailand through its website, free of charge [29]. Given that community pharmacists may be unaware of this fact, promotion and communication to pharmacists on this website should be increased. 

Consistent with several other studies [10,18,20,21,25,26,27], this study found that lack of time was perceived to be a significant barrier to providing smoking cessation services. Interestingly, lack of time was more likely to be perceived as a barrier among those community pharmacists providing smoking cessation than those that did not. Further investigation should examine the reasons for providing smoking cessation services among pharmacists, even when they perceived time constrain was a barrier. 

Apart from perceived barriers reported by the pharmacists, motivation to provide smoking cessation services was investigated. This study found that the motivation of community pharmacists in providing smoking cessation services was mainly to enhance the pharmacy profession in helping patients, smokers, individuals, society, and the nation, to be free from the dangers of tobacco. It also expanded the roles of community pharmacies in tobacco control and smoking cessation. In addition, community pharmacists could create awareness of the dangers of smoking and be willing to communicate with patients and smokers to help them to quit smoking, and prevent diseases caused by smoking. This study showed that the motivation for providing public health services was related to the pharmacy profession and not a financial issue, particularly considering those working in independent pharmacies. Similar to one study conducted in the UK [9], professional responsibility was the motivation for providing smoking cessation services in the community pharmacy. Financial issues were not the reason, because these cessation services at the community pharmacy were provided free of charge, unless smokers needed to use pharmacotherapy to stop smoking. 

In Thailand, the TPNTC plays a vital role in motivating and providing training to pharmacists to be readily equipped with knowledge and skills for smoking cessation services. This study also confirmed that being a member of the TPNTC encouraged pharmacists to serve greater roles in helping smokers to quit. However, there remains a need to further motivate and encourage community pharmacists to continue their smoking cessation roles, and to expand and communicate with other community pharmacists to initiate smoking cessation services in their community pharmacies, as well as to overcome perceived barriers preventing them from providing smoking cessation services. 

### 4.3. Limitations and Strengths

Some limitations should be acknowledged. First, the presented data were not representative of all community pharmacists in Thailand due to the survey’s very low response rate of 7.9% (selection bias, overestimation). Although the study received a low response rate from the respondents, the responses were received from all throughout Thailand: Bangkok, North, Central, Northeast, and the south. In addition, participants who returned the questionnaire showed their interest in promoting the pharmacy profession concerning tobacco control, suggesting their opinions and practices were crucial. Another reason for the low response rate could be that they did not perform smoking cessation services, so they were uninterested in sharing their opinions. In addition, most community pharmacists in Thailand did not have assistants; thus, they did not have time to complete the questionnaire. Follow-up telephone calls were made to increase the survey response rate. Unfortunately, some telephone numbers were wrong. However, this low response rate was similar to a study conducted with community pharmacists using an online-questionnaire in Australia, with a response rate of 6% [15]. Second, given that the survey collected self-reported data, some measurement errors are likely. For example, the Table 4 item, “Smokers can quit or decrease smoking” depends on self-reports of pharmacists. Therefore, pharmacists could overestimate their success of providing smoking cessation services. 

However, this study collected information from community pharmacists across Thailand. It constitutes a countrywide survey regarding the roles of community pharmacists in Thailand, in providing smoking cessation services to the public. Although the low response rate implied caution in interpreting the findings and generalizing of the results, the findings are still legitimate in reflecting the situation of the roles of community pharmacists in tobacco control and smoking cessation, and highlighting pharmacy professionals’ need to extend smoking cessation services. 

## 5. Conclusions

In conclusion, only a minority of community pharmacists in Thailand are engaged in smoking cessation activities, even though some perceived barriers existed. Community pharmacists who provided smoking cessation services were more likely to have pharmacist assistants, be a member of the TPNTC, and have more than one pharmacist on duty. Being unable to follow-up and inadequate staffing were the top two perceived barriers. Those pharmacists not providing smoking cessation services perceived five barriers more than those providing the services, i.e., lack of demand, no smoking cessation products available, lack of knowledge and skills, lack of self-confidence, and lack of media/equipment for providing smoking cessation services. 

Actions to improve smoking cessation services in community pharmacies in Thailand could be implemented through the TPNTC or pharmacy faculties of each concerned university. For example, the TPNTC or the universities could provide short course trainings on smoking cessation services to practicing pharmacists, especially those who did not receive relevant training at undergraduate or graduate levels. As the educational materials or media/equipment to aid smoking cessation services could be requested from the ASH Thailand for free, their availability should be communicated to pharmacists. At universities, pharmacy students should be encouraged to practice the delivery of smoking cessation services through their clerkships in year 6. Finally, a health promotion campaign targeting smokers could advertise the availability of community pharmacists’ smoking cessation services. 

## Figures and Tables

**Table 1:** Participants’ characteristics in providing smoking cessation services (*n* = 413).

Characteristic		Total (*n* = 413)	Pharmacists Providing Smoking Cessation Services (*n* = 152)	Pharmacists not Providing Smoking Cessation Services (*n* = 261)	*p*-Value
Sex					
	Male	171 (41.4)	59 (38.8)	112 (42.9)	0.469
	Female	242 (58.6)	93 (61.2)	149 (57.1)	
Age (years)					
	<30	92 (22.4)	48 (31.8)	44 (17.0)	0.009
	30–39	120 (29.3)	38 (25.2)	82 (31.7)	
	40–49	95 (23.2)	31 (20.5)	64 (24.7)	
	>49	103 (25.1)	34 (22.5)	69 (26.6)	
	mean ± SD	40.9 ± 12.7	38.7 ± 12.0	42.2 ± 13.0	0.007
Being a pharmacist (years)					
	<10	190 (46.6)	74 (49.0)	116 (45.1)	0.759
	10–19	120 (29.4)	43 (28.5)	77 (30.0)	
	>19	98 (24.0)	34 (22.5)	64 (24.9)	
Having pharmacist assistant					
	No	144 (34.9)	37 (24.3)	107 (41.0)	0.001
	Yes	269 (65.1)	115 (75.7)	154 (59.0)	
Number of pharmacists on duty					
	1	284 (68.8)	76 (50.0)	208 (79.7)	<0.001
	>1	129 (31.2)	76 (50.0)	53 (20.3)	
Location of pharmacy					
	Bangkok	126 (30.7)	48 (31.6)	78 (30.1)	0.733
	North	110 (26.8)	41 (27.0)	69 (26.6)	
	Central	89 (21.6)	29 (19.1)	60 (23.2)	
	Northeast	45 (10.9)	20 (13.2)	25 (9.6)	
	South	41 (10.0)	14 (9.2)	27 (10.4)	
Location of pharmacy					
	Shopping center	46 (12.1)	21 (15.6)	25 (10.2)	0.141
	Community	333 (87.9)	114 (84.4)	219 (89.8)	
Membership in TPNTC					
	Yes	115 (27.9)	93 (61.2)	22 (8.4)	<0.001
	No	298 (72.1)	59 (38.8)	239 (91.6)	
Smoking cessation products available					
	Yes	316 (76.5)	145 (95.4)	171 (65.5)	<0.001
	No	97 (23.5)	7 (4.6)	90 (34.5)	
Providing smoking cessation is a role of community pharmacists					
	Yes	400 (96.9)	152 (100.0)	248 (95.0)	0.003
	No	13 (3.1)	0 (0.0)	13 (5.0)	

Note: TPNTC, Thai Pharmacy Network for Tobacco Control. Percentages presented are based on numbers of respondents answering each question.

**Table 2:** Smoking cessation products provided at the community pharmacy.

Smoking Cessation Products	Total (*n* = 413)	Pharmacists Providing Smoking Cessation Services (*n* = 152)	Pharmacists not Providing Smoking Cessation Services (*n* = 261)	*p*-Value
Nicotine gu	300 (72.6)	140 (92.1)	160 (61.3)	<0.001
Bupropion	111 (26.9)	75 (49.3)	36 (13.8)	<0.001
Nicotine patch	81 (19.6)	54 (35.5)	27 (10.3)	<0.001
Herbal mixture for smoking cessation	72 (17.4)	43 (28.3)	29 (11.1)	<0.001
Nortriptyline	56 (13.6)	41 (26.9)	15 (5.7)	<0.001
Varenicline	10 (2.4)	8 (5.3)	2 (0.8)	0.006
Others	42 (10.2)	29 (19.1)	13 (5.0)	<0.001

Note: Percentages presented are based on numbers of respondents answering each question.

**Table 3:** Activities of smoking cessation services (*n* = 147).

Activities	*n* (%)
Providing materials for smoking cessation (*n* = 147)	110 (74.8)
Brochures (*n* = 110	87 (79.1)
Video (*n* = 110)	5 (4.6)
Poster (*n* = 110)	85 (77.3)
Showing symbols in front of pharmacy (*n* = 147)	105 (71.4)
A designated area for smoking cessation (*n* = 147)	53 (36.0)
Engaging with the community (*n* = 147)	50 (34.0)

**Table 4:** Number of smokers receiving smoking cessation services and time spent on smoking cessation services (*n* = 152).

	*n*	Mean (±SD)	Median (Q1, Q3)	Min	Max
Number of smokers receiving smoking cessation services (person/month)					
Total number of smokers receiving smoking cessation service (person/month)	135	3.4 ± 4.6	2 (1, 3)	0	30
Smokers can quit smoking (person/pharmacy)	90	6.4 ± 6.9	4 (2, 10)	0	40
Smokers can decrease smoking (person/pharmacy)	76	9.0 ± 9.0	5 (3, 10)	0	40
Time for providing smoking cessation service (min/person)					
The first service	141	15.1 ± 10.9	10 (5, 20)	1	60
Each follow-up visit	129	8.9 ± 6.7	5 (5, 10)	1	30

**Table 5:** Perceived barriers on providing smoking cessation services (*n* = 413).

	Total (*n* = 413)	Pharmacists Providing Smoking Cessation Services (*n* = 152)	Pharmacists not Providing Smoking Cessation Services (*n* = 261)	*p*-Value
1. Unable to follow-up	346 (84.6)	122 (81.9)	224 (86.1)	0.258
2. Inadequate staffing	299 (73.5)	111 (74.5)	188 (72.9)	0.816
3. Lack of time	262 (64.1)	115 (76.2)	147 (57.0)	<0.001
4. Lack of population demand	286 (69.8)	87 (57.6)	199 (76.8)	<0.001
5. No smoking cessation products	126 (30.7)	26 (17.2)	100 (38.5)	<0.001
6. Lack of knowledge and skills	216 (52.9)	64 (43.0)	152 (58.7)	0.003
7. Lack of self-confidence	166 (40.7)	48 (32.0)	118 (45.7)	0.007
8. Lack of media/equipment	299 (73.3)	102 (67.5)	197 (76.6)	0.049
9. No appropriate place	199 (48.7)	68 (45.0)	131 (50.8)	0.305
10. No motivation	177 (43.7)	64 (43.0)	113 (44.1	0.836
11. No payment	95 (23.4)	41 (27.5)	54 (21.0)	0.146

Note: Percentages presented are based on numbers of respondents answering each question.
